# Low *ABCB1* Gene Expression Is an Early Event in Colorectal Carcinogenesis

**DOI:** 10.1371/journal.pone.0072119

**Published:** 2013-08-19

**Authors:** Vibeke Andersen, Ulla Vogel, Sine Godiksen, Franz B. Frenzel, Mona Sæbø, Julian Hamfjord, Elin Kure, Lotte K. Vogel

**Affiliations:** 1 Medical Department, Hospital of Southern Jutland, Aabenraa, Denmark; 2 Institute of Regional Health Research, University of Southern Denmark, Odense, Denmark; 3 Medical Department, Regional Hospital Viborg, Viborg, Denmark; 4 National Research Centre for the Working Environment, Copenhagen, Denmark; 5 Department of Cellular and Molecular Medicine, University of Copenhagen, Copenhagen, Denmark; 6 Telemark University College, Faculty of Arts and Sciences, Department of Environmental and Health Studies, Telemark, Norway; 7 Department of Genetics, Institute for Cancer Research, Oslo University Hospital, Oslo, Norway; Sun Yat-sen University Cancer Center, China

## Abstract

The *ABCB1/MDR1* gene product ABCB1/P-glycoprotein is implicated in the development of colorectal cancer (CRC). *NFKB1* encodes transcription factors regulating expression of a number of genes including *ABCB1*. We have previously found association between the *ABCB1* C-rs3789243-T polymorphism and CRC risk and interactions between the *ABCB1* C-rs3789243-T and C3435T polymorphisms and meat intake in relation to CRC risk (Andersen, BMC Cancer, 2009, 9, 407). *ABCB1* and *NFKB1* mRNA levels were assessed in intestinal tissue from 122 CRC cases, 101 adenoma cases (12 with severe dysplasia, 89 with mild-moderate dysplasia) and from 18 healthy individuals, together with gene polymorphisms in *ABCB1* and *NFKB1. ABCB1* mRNA levels were highest in the healthy individuals and significantly lower in mild/moderate and severe dysplasia tissue (*P*<0.05 for both), morphologically normal tissues close to the tumour (P<0.05), morphologically normal tissue at a distance from the tumour (P<0.05) and CRC tissue (*P*<0.001). Furthermore, *ABCB1* mRNA levels were lower in adenomas and carcinomas compared to morphologically normal tissue from the same individuals (*P*<0.01). The *ABCB1* C-rs3789243-T and *NFKB1* -94ins/del homozygous variant genotypes were associated with low *ABCB1* mRNA levels in morphologically normal sigmoid tissue from adenoma cases (*P*<0.05 for both). *NFKB1* mRNA levels were lower in both tumour and normal tissue from cancer patients (*P*<0.001) as compared to healthy individuals but we were unable to show association between *NFKB1* -94ins/del genotype and *NFKB1* mRNA levels. This study suggests that low *ABCB1* mRNA levels are an early event in CRC development and that the two polymorphisms affect *ABCB1* mRNA levels whereas low *NFKB1* mRNA levels occur later in carcinogenesis. Low ABCB1 protein levels may promote colorectal carcinogenesis through increasing intracellular exposure to carcinogenic ABCB1 substrates.

## Introduction

Colorectal cancer (CRC) constitutes the second most common cancer in the Western world and the prevalence is expected to increase due to demographic trends and adaption to westernized lifestyle in developing countries. In the Western world, one in 20 will develop CRC before the age of 75.

ABCB1/P-glycoprotein is a membrane protein, encoded by *ABCB1/MDR1,* which transports substrates from the enterocytes to the intestinal lumen, thereby, restricting the exposure of the enterocytes to the substrates of the ABCB1 transporter. Known substrates include various xenobiotics and endogenous compounds such as cholesterol, IL-1β, IL-2, IL-4, and IFNγ [1]. The ABCB1 has been implicated in intestinal carcinogenesis in animal models and in epidemiological studies. Abcb1/mdr1a knock-out mice develop colitis and later intestinal adenocarcinomas [2] suggesting that the absence of ABCB1 confers risk of inflammation-related CRC. In human, *ABCB1* polymorphisms are associated with risk of CRC in some but not all studies [3]–[6]. The *ABCB1* intron 3 C-rs3789243-T is a marker polymorphism and was found to be associated with risk of CRC in a prospective, population-based study (OR = 1.52, 95%CI 1.12–2.06) [3]. Similarly, a meta-analysis of case-control studies found an increased risk of CRC among carriers of the combined C3435T and G2677T/A wildtype alleles in Caucasians (OR = 1.22, 95%CI 1.03–1.44). In a subsequent study, the C3435T polymorphism was assessed in three large cohorts [5]. The C3435T C-allele was associated with a low risk of CRC (OR = 0.79, 95%CI 0.66–0.96) in the cohort from Southwest Germany (1809 cases) and a high risk, although not statistically significant, in the cohort from Northern Germany (2169 cases) whereas no change of risk was found in the Czech cohort (699 cases) [5].

*ABCB1* levels in solid tumours have been determined in a few studies [7]–[9]. ABCB1 protein levels were found to be low in CRC tissue compared to well-differentiated tissue in archival material from 51 cancer patients estimated by immunohistochemistry [8]. Also, *ABCB1* mRNA and protein levels were found to be lowered in renal cell carcinoma tissue compared to normal looking cortex in 82 nephrectomised cancer patients [7]. In the German study, the variant alleles of C2677T/A, and G3435T polymorphisms were associated with high *ABCB1* mRNA levels in normal renal tissue [7]. In contrast, the variant alleles of 2677T and 3435T polymorphisms were associated with low mRNA levels in 73 normal liver tissue sample from Chinese patients [10]. Although intestinal ABCB1 transport activity can be determined using a model substrate [11], this has not been done on cancer cells or cancerous tissue.

The *MDR1* gene has a TATA box-less promoter regulated by a large number of factors including pregnane X receptor (PXR) and nuclear factor-κB (NF-κB). The *NFKB1* -94ins/del polymorphism is a four nucleotide insertion/deletion polymorphism in the promoter region of *NFKB1* leading to lower transcription levels and lower protein levels of the NF-κB subunits p50 and p105 [12]. Genetically determined low NF-κB p50 levels were associated with an increased risk of CRC among Danes, whereas genetic variation in PXR was not associated with CRC risk [13].

In the present study our aim was to assess the intestinal levels of *ABCB1* and *NFKB1* mRNA in adenoma and CRC cases and in control subjects in order to characterize the role of ABCB1 in the development of CRC. Furthermore, we assessed the impact of genetic variants in *ABCB1* and *NFKB1* on the *ABCB1* mRNA levels in morphologically normal and affected colon tissues.

## Materials and Methods

### Study Cohort

The KAM (Kolorektalkreft, arv og miljø) cohort is based on the screening group of the Norwegian Colorectal Cancer Prevention study (the NORCCAP study) in the county of Telemark and a series of clinical CRC cases operated at Telemark Hospital (Skien) and Ulleval University Hospital (Oslo) [14], [15]. In short, 20,780 healthy men and women, 50–64 years of age, drawn at random from the population registry in Oslo (urban) and the county of Telemark (mixed urban and rural) were invited to have a flexible sigmoidoscopy screening examination. The KAM cohort is based on an ethnically homogeneous group of Norwegian origin.

The KAM biobank consists of samples from individuals with adenomas in the large intestine (991 adenomas and 53 hyperplastic polyps), 234 cases with CRC and 400 controls, defined as individuals with normal findings at flexible sigmoidoscopy screening. The study was performed in accordance with the Helsinki Declaration. The Regional Ethics Committee and the Data Inspectorate approved the KAM study (S-98190, 2009/2021). The ID number for the study is NCT00119912 at ClinicalTrials.gov. All participants gave verbal and written informed consent.

### Biological Material

In the present study, blood samples were available from 167 cases with carcinomas, 990 cases with adenomas and 400 controls. Intestinal tissue was available from 121 cases with carcinoma, 100 cases with adenomas and 18 controls with normal endoskopic findings. From individuals with adenomas, control tissue was sampled 30 cm above the anus. From patients with carcinomas, two samples of morphologically normal tissue were taken from the surgical specimen. One sample was taken adjacent to the cancer (normal tissue 2) and the other sample was taken as distant from the cancer as possible (normal tissue 1). Complete samples were available from 75 cases with mild-moderate dysplasia, 11 cases with severe dysplasia, and 99 CRC cases. The histology of the adenomas was examined independently by two histopathologists, who categorised the degree of dysplasia as either mild/moderate (n = 87) or severe (n = 13). Consensus was reached in all cases. Carcinomas were classified according to Dukes staging.

### Real-time Reverse Transcriptase Polymerase Chain Reaction

The tissue samples were frozen as soon as possible after surgery and stored in liquid nitrogen until RNA purification. Total RNA was purified from tissue as recommended by the manufacturer using E.Z.N.A. Total RNA Kit II (cat no. R6834-02, Omega Bio-Tek) and the RNase Free DNase kit I (cat. no. E1091-01). cDNA synthesis was performed on approximately 200 ng RNA per 20 µl using the High-Capacity cDNA Archive Kit (part.no. 4375222, Applied Biosystems). Quantitative real time RT-PCR for *ABCB1* was performed on the ABI7300 sequence detection system (Applied Biosystems) in Universal PCR Master Mix (part.no 4326614, Applied Biosystems) using 240 nM probe and 200 nM primers. Primers and probe were: *ABCB1* forward 5′-CTC AGA CAG GAT GTG AGT TGG TTT-3′; *ABCB1* reverse 5′-CTT GGA ACC TAT AGC CCC TTT AA-3′; *ABCB1* probe 5′- FAM-ACC ACT GGA GCA TTG ACT ACC AGG C-BHQ-3′. NFKB1: forward primer: 5′-CAC GAA TGA CAG AGG CGT GTA-3′; reverse: 5′-GGA TTA GCT CTT TTT CCC GAT CT-3′; NFKB1-probe: 5′-FAM-CTC TTG GTG CAC CCT GAC CTT GCC-BHQ-3′.

Primers were designed within different exons and with the probe covering an exon-exon border to prevent amplification of genomic DNA (*ABCB1*) or with a primer covering an exon-exon border (*NFKB1*). Primers and probes were obtained from TAGCopenhagen (Denmark). The endogenous *β-actin* control was obtained using a pre-developed assay (part.no.4310881E) from Applied Biosystems. In a validation experiment, a dilution series was assayed by the comparative C_t_ method [16]. The assays were quantitative over a range of 128-fold dilution. Samples were quantified in triplicates. The standard deviation of triplicates was 6% or less. The standard deviation on repeated measurements of the same sample (the control) in separate experiments was 16% (*ABCB1*) and 13% (*NFKB1*), indicating the day-to-day variation of the assay. Negative controls (where the RNA was not converted into cDNA) and positive controls were included in all runs. Samples for which either the *β-actin, ABCB1,* or *NFKB1* values fell outside the upper or lower limits of the standard curve were excluded from the study.

### Genotyping

Genomic DNA was isolated from blood samples according to standard procedures. *ABCB1* C-rs3789243-T genotyping data was retrieved from Andersen *et. al*. [6]. *ABCB1* C3435T (rs1045642) and *NFKB1* -94ins/del (rs28362491), were genotyped by KBioscience (kbioscience.co.uk). For *ABCB1,* C was considered as the wildtype allele even though it was not the major allele, since this is the commonly used nomenclature.

### Statistics

GraphPad Prism 4 was used for the statistic calculations. The data were not adjusted for gender since the incidence ratio of CRC between the genders is 1∶1 in Norway. Kruskal Wallis and Dunńs Multiple Comparison test was used to compare the mRNA level of all intestinal tissues and Paired Student’s t-test was used for comparison of affected tissue and morphological normal tissue from the same individual.

## Results

### *ABCB1* mRNA Levels in Intestinal Tissue

**Figure 1:**
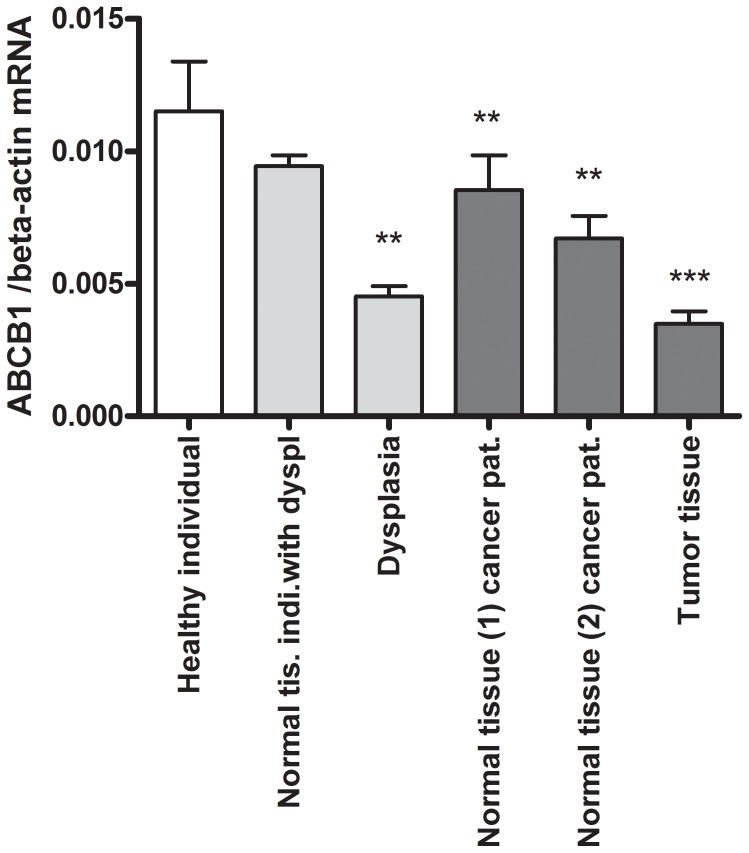
*ABCB1* mRNA levels in morphologically normal and affected tissues normalised to the *β-actin* mRNA level. *P-value <0.05, **P-value <0.01, ***P-value <0.001.

Study participants are described in Table 1. *ABCB1* mRNA levels were significantly lower in dysplastic tissue (P<0.01), morphologically normal tissues 1 and 2 from cancer patients (P<0.01, for both) and tumour tissue (P<0.001) compared to healthy individuals (Figure 1). In order to elucidate whether *ABCB1* mRNA levels are diminished as an early event in colorectal carcinogenesis, individuals with adenomas were further subdivided into mild/moderate dysplasia (N = 89) and severe dysplasia (N = 12) (Table 2). Table 2 shows a low level of *ABCB1* mRNA already in mild/moderate dysplasia which is maintained at a low level throughout carcinogenesis. Comparing *ABCB1* mRNA levels, a significantly lower mRNA level was found in the mild/moderate adenoma, severe adenoma and CRC tissues (Table 2) as compared to morphologically normal tissue from the same individuals. There was no correlation between the *ABCB1* mRNA levels and age, gender or Duke’s stage of the carcinoma (Dukes stage A (n = 19), stage B (n = 47), and stage C (n = 29)).

**Table 1:** Characteristics of study participants.

	Controls^3^	Individuals with Mild/moderate Dysplasia	Individuals withSevere Dysplasia	Individuals with Carcinomas
Total number of persons in the study	18	89	12	122
Number of men/women	6/12	63^1^/26	6/6	62^1^/53
Mean age ± SD	56.8±4.5	56.9±3.6	55.1±3.1	69.4±11.9^2^

1 There were significantly more males in the group with mild dysplasia and colorectal cancer compared to the group of healthy individuals (p<0.001, Chi squared test).

2 The age is significant higher among patients with CRC compared to the three other groups (Kruskal-Wallis and Dunn's Multiple Comparison test, p<0.001).

3 Normal endoscopy.

**Table 2:** *ABCB1* mRNA levels in morphologically non-affected and affected tissues normalised to the *β-actin* mRNA level.

Variable	mRNA level inmorphologically normaltissues Mean ± S.D.	P^a^	mRNA level inadenomas/carcinomasMean ± S.D.	P^a^	P^b^
Controls	0.012±0.008				
Individuals withmild/moderate dysplasia	0.009±0.004	NS	0.005±0.004	<0.05	<0.001
Individuals withsevere dysplasia	0.009±0.03	NS	0.003±0.002	<0.05	<0.001
Cancer patients	0.009±0.014 (normal tissue 1)0.007±0.009 (normal tissue 2I)	<0.05<0.05	0.003±0.005“	<0.001	<0.001<0.01

NS = not significant.

a p-value for the comparison to the expression levels in tissue from healthy individuals using Kruskal Wallis and Dunńs Multiple Comparison test.

b p-value for the comparison of the expression levels in normal and affected tissue from the same individual using Paired Student’s t-test.

### Genetic Polymorphisms and *ABCB1* mRNA Levels

**Figure 2:**
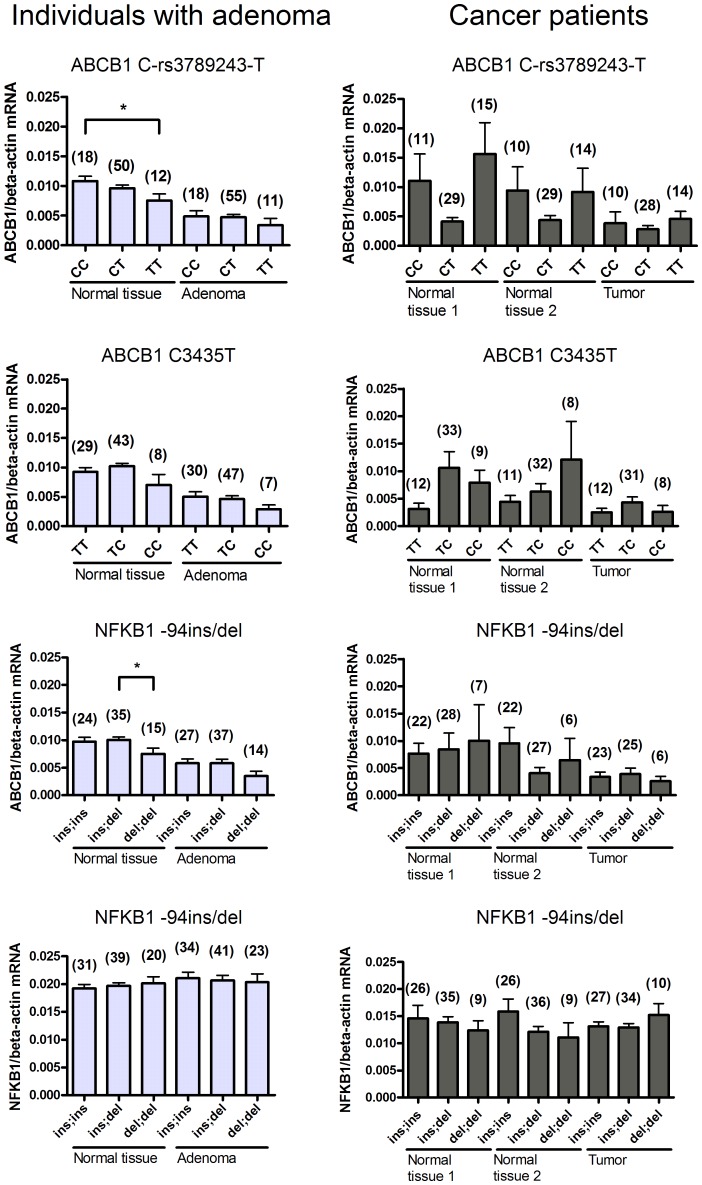
Relation between *ABCB1* C-rs3789243-T, C3435T and *NFKB1* -94ins/del polymorphisms and *ABCB1* mRNA level in morphologically normal and affected intestinal tissue from individuals with adenomas (left panel) and CRC (right panel). The number of individuals with each genotype is indicated in brackets above the column. *P-value <0.05.

Figure 2 shows *ABCB1* mRNA levels in intestinal adenoma cases (left panel) and carcinoma cases (right panel) subdivided by *ABCB1* C-rs3789243-T, C3435T, and *NFKB1* -94ins/del genotypes, respectively. In morphologically normal sigmoid tissue from the intestinal adenoma cases, significantly lower *ABCB1* mRNA levels were found for carriers of the *ABCB1* C-rs3789243-T variant homozygous genotype compared to the homozygous wildtype carriers (*P<*0.05). Furthermore, the *NFKB1* del/del genotype was associated with low *ABCB1* mRNA level compared with the heterozygous genotype (*P<*0.05). Similar trends, however not statistically significant, were found in adenoma tissue. Among carcinoma cases, no associations between any genotypes and *ABCB1* mRNA levels were found in any tissue (Figure 2, right panel).

These data suggest that NFKB1 play a role in regulation of the expression of *ABCB1* gene in normal tissue as represented by the morphological normal sample from individuals with dysplasia but not in cancerous tissue or normal tissue from cancer patients. The *NFKB1* mRNA levels were therefore determined in all intestinal samples in order to further investigate this.

### *NFKB1* mRNA Levels in Intestinal Tissue

**Figure 3:**
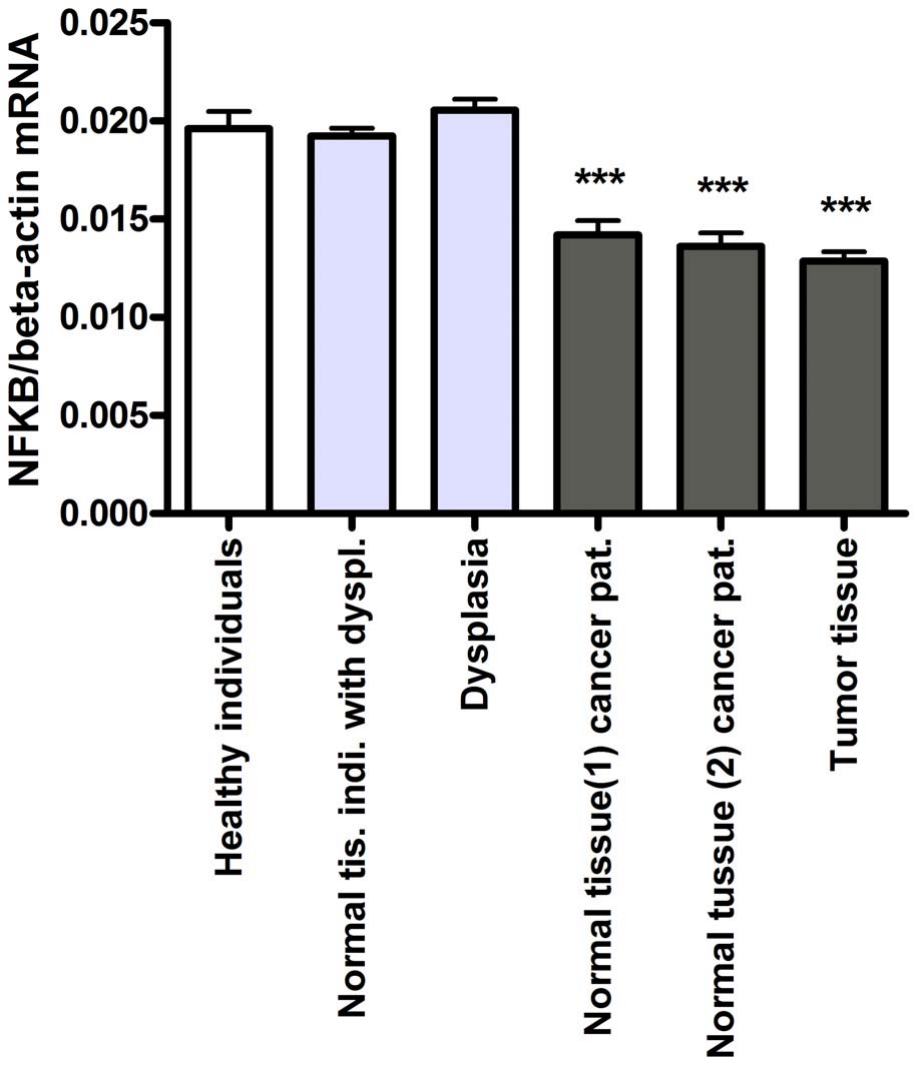
*NFKB1* mRNA levels in morphologically normal and affected tissues normalised to the *β-actin* mRNA level. ***P-value <0.001.

Generally the *NFKB1* mRNA levels were significantly lower in tissues from cancer patients but unaffected in individuals with dysplasia. *NFKB1* mRNA levels were significantly lower in morphologically normal tissues 1 and 2 from cancer patients, and tumour tissue (P<0.001 for all) compared to healthy individuals (Figure 3). We found no difference between affected and non-affected tissue in individuals with dysplasia or cancer patients (Table 3). There was no correlation between the *NFKB1* mRNA levels and age, gender, or Duke’s stage of the carcinoma.

**Table 3:** *NFKB1* mRNA levels in morphologicallynon-affected and affected tissues normalised to the *β-actin* mRNA level.

Variable	mRNA level inmorphologically normaltissues ± S.D.	P^a^	mRNA level inadenomas/carcinomasMean ± S.D.	Mean P^a^	P^b^
Controls	0.020±0.004				
Individuals withmild/moderate dysplasia	0.019±0.004	NS	0.021±0.006	NS	NS
Cancer patients	0.014±0.008 (normal tissue 1)0.014±0.008 (normal tissue 2)	<0.001<0.001	0.013±0.005“	<0.001	NSNS

NS = not significant.

a P-value for the comparison to the expression levels in tissue from healthy individuals using Kruskal Wallis and Dunńs Multiple Comparison test.

b P-value for the comparison of the expression levels in normal and affected tissue from the same individual using Paired Student’s t-test.

### Genetic Polymorphism and *NFKB1* mRNA Levels

Figure 2 lower panel shows *NFKB1* mRNA levels in intestinal adenoma cases (left panel) and carcinoma cases (right panel) subdivided by the *NFKB1* -94ins/del genotype. We found no significant difference in the *NFKB1* mRNA level depending on the NFKB1 -94ins/del genotype.

## Discussion

In the present study, we found that intestinal tissue from healthy individuals had the highest *ABCB1* mRNA level. Apparently morphologically normal tissue from cancer cases had significantly but only slightly lowered *ABCB1* mRNA levels. In contrast, the mRNA levels in both adenoma tissue and carcinoma tissue were decreased by half compared to the mRNA level found in the healthy controls. *ABCB1* mRNA levels were also lower in adenomas and carcinomas compared to morphologically normal tissue from the same individuals (Table 2).

Our findings on carcinomas are in agreement with previous findings by Judicibus *et al.* on CRC and Haenisch *et al.* on renal cell carcinomas, who both found low *ABCB1* mRNA and/or protein levels in tumour tissue compared to non-affected tissue surrounding the tumour (10;11). We extend the study by Judicibus *et al.*, by analysing tissue from healthy individuals and individuals with adenomas.

The presence of a reduced *ABCB1* mRNA level already in mild/moderate dysplastic tissue (Table 2) suggests that lowered *ABCB1* mRNA level is an early event in colorectal carcinogenesis. Therefore, assuming that a lowered level of *ABCB1* mRNA results in low ABCB1 activity in the adenomas, this would lead to a higher intracellular exposure to various carcinogenic substrates of the ABCB1 transporter. The finding that once CRC has developed, low *ABCB1* mRNA levels were found in both carcinoma tissue and morphologically normal tissue surrounding the tumour, could suggest that low *ABCB1* mRNA precede tumour formation or that the presence of tumour tissue affects gene expression in the surrounding tissue.

We evaluated the association between genetic variants in *ABCB1* and *NFKB1*, and intestinal *ABCB1* mRNA levels. *ABCB1* C-rs3789243-T and *NFKB1* -94ins/del homozygous variant genotypes were associated with lowered *ABCB1* mRNA levels in morphologically normal tissue from adenoma cases. The *ABCB1* gene is highly polymorphic and extensive linkage between polymorphisms make identification of causal variants difficult [17]. Thus, it is most likely that the polymorphism is biologically non-functional and the detected association with *ABCB1* mRNA levels is caused by linkage to one or more functional polymorphisms [17]. The studied *NFKB1* ATTG ins/del polymorphism is a functional deletion in the promoter region of *NFKB1* encoding the p50 and p105 subunits of NF-κB. The deletion abolishes a transcription factor binding site in the promoter region and leads to lower NF-κB p50 synthesis [12]. However, we found no association between *NFKB1* ins/del genotype and *NFKB1* mRNA levels. It seems contradictory that we are able to find association between the *NFKB1* -94ins/del polymorphism and *ABCB1* mRNA levels but not with *NFKB1* mRNA levels. The reason might be that even relatively small differences in p50 protein levels may be sufficient to change transcription rates of target genes as shown by Kaban *et al.* [12]. Alternatively, it may be due to the relative low number of individuals analysed. Transcription of C-reactive protein is known to be activated by the NF-κB p50 dimer and the *NFKB1* ins/del polymorphism was previously shown to be associated with blood levels of C-reactive protein in a study group of more than 1800 women [18].

Furthermore, our results suggests that the observed low *ABCB1* mRNA level in adenoma tissue must be caused by changes is the concentration of transcription factors other than NF-κB p50 as *NFKB1* mRNA levels was unchanged in adenoma tissue.

In individuals with adenomas, we found that carriers homozygous for the *ABCB1* C-rs3789243-T and *NFKB*-94 deletion variant alleles have significantly lower *ABCB1* mRNA levels in morphologically normal tissue suggesting that these polymorphisms cause lower *ABCB1* mRNA levels. In contrast, *ABCB1* mRNA levels in the carcinogenic tissue and the morphologically normal tissue surrounding the tumour are lowered independently of the studied polymorphisms in *ABCB1* and *NFKB1* (Figure 1 and 2) suggesting any regulation by these polymorphisms is lost once adenomas have been formed.

In a prospective Danish study, carriers of the *NFKB1* -94 deletion and the *ABCB1* intron 3 C-rs3789243-T variant alleles were at 1.45 and 1.55-fold higher risk of CRC, respectively, than carriers of the corresponding homozygous wildtype (95%CI 1.10–1.92, *P* = 0.03 and 95%CI 1.12–2.06, *P = *0.03, respectively) [3], [13]. In the same studies, interactions between *ABCB1* and *NFKB1* polymorphisms and intake of red and processed meat in relation to CRC risk were found [3], [13]. *ABCB1* C3435T homozygous C-allele carriers, *ABCB1* C-rs3789243-T T-allele carriers and *NFKB1* -94 deletion carriers were at high risk of CRC by meat intake (*P* for interaction = 0.02, 0.01, and 0.03, respectively).

At present, it is not clear how meat intake interacts with *ABCB1* in relation to CRC development [19]. There are no known dietary carcinogens identified as ABCB1 substrates. Studies of abcb1 knock-out mice strongly suggest that abcb1 deficiency drives an inflammation-related carcinogenesis. The same mechanism may be important in human which is suggested by the identification of *ABCB1* as a ‘risk gene’ in inflammatory bowel diseases [20].

This study used a case-control design. Because our hypothesis was biologically based, we did not correct for multiple analyses [21].A main strength of the study is the relatively large sample size. However, in the light of the obtained P-values and the number of statistical tests performed, we cannot exclude that some of our positive findings may be due to chance.

In conclusion, this study suggests that low *ABCB1* mRNA expression is an early event in CRC development. *ABCB1* mRNA levels are associated with genetic variation in *ABCB1*. Furthermore, low levels of *NFKB1* encoding NFκB p50, which is involved in regulation of *ABCB1* expression is a late event in carcinogenesis. Low *ABCB1* levels may promote CRC by increasing intracellular exposure to carcinogenic or inflammatory ABCB1 substrates. Further studies on the functional role of *ABCB1* in carcinogenesis are warranted.
