# Correction: Low *ABCB1* Gene Expression Is an Early Event in Colorectal Carcinogenesis

**DOI:** 10.1371/annotation/7cd6c6a6-b281-4bf3-b478-a289ed39b375

**Published:** 2013-09-12

**Authors:** Vibeke Andersen, Ulla Vogel, Sine Godiksen, Franz B. Frenzel, Mona Sæbø, Julian Hamfjord, Elin Kure, Lotte K. Vogel

There were errors in the Author Contributions section. The correct version of this section is available below.

Conceived and designed the experiments: VA UV EK LKV. Performed the experiments: FBF MS EK LKV, Analyzed and interpreted the data: VA UV SG JH EK LKV. Contributed reagents/materials/analysis tools: MS JH EK. Wrote the paper: VA UV LKV EK. Rose the funding: VA EK. 

